# Deregulated MicroRNAs in Biliary Tract Cancer: Functional Targets and Potential Biomarkers

**DOI:** 10.1155/2016/4805270

**Published:** 2016-11-09

**Authors:** Christian Mayr, Marlena Beyreis, Andrej Wagner, Martin Pichler, Daniel Neureiter, Tobias Kiesslich

**Affiliations:** ^1^Laboratory for Tumour Biology and Experimental Therapies (TREAT), Institute of Physiology and Pathophysiology, Paracelsus Medical University, Salzburg, Austria; ^2^Department of Internal Medicine I, Paracelsus Medical University/Salzburger Landeskliniken (SALK), Salzburg, Austria; ^3^Division of Oncology, Department of Internal Medicine, Medical University of Graz, Graz, Austria; ^4^Institute of Pathology, Paracelsus Medical University/Salzburger Landeskliniken (SALK), Salzburg, Austria

## Abstract

Biliary tract cancer (BTC) is still a fatal disease with very poor prognosis. The lack of reliable biomarkers for early diagnosis and of effective therapeutic targets is a major demanding problem in diagnosis and management of BTC. Due to the clinically silent and asymptomatic characteristics of the tumor, most patients are diagnosed at an already advanced stage allowing only for a palliative therapeutic approach. MicroRNAs are small noncoding RNAs well known to regulate various cellular functions and pathologic events including the formation and progression of cancer. Over the last years, several studies have shed light on the role of microRNAs in BTC, making them potentially attractive therapeutic targets and candidates as biomarkers. In this review, we will focus on the role of oncogenic and tumor suppressor microRNAs and their direct targets in BTC. Furthermore, we summarize and discuss data that evaluate the diagnostic power of deregulated microRNAs as possible future biomarkers for BTC.

## 1. Introduction to Biliary Tract Cancer and MicroRNAs

Biliary tract cancer (BTC) is a malignant disease of the biliary tract epithelia cells, the cholangiocytes. Depending on the localization of the tumor, the term BTC comprises cholangiocarcinomas (CC) of the intrahepatic (IHC) and extrahepatic (EHC) bile ducts, as well as gallbladder cancer (GBC) [1, 2]. The most common BTC is GBC, whereas, for CCs, approximately two-thirds involve the extrahepatic bile ducts [3, 4]. In general, BTC is a rare disease with 3–8 new cases per 100,000 population in the US. However, CC is the second most common hepatic malignancy after hepatocellular carcinoma and the incidence of CC has increased over the last years [5, 6]. Epidemiologic studies revealed strong regional differences in development of BTC, with the area of Khon Kaen in Thailand being the most drastic example, where CC accounts for over 85% of all cancers [3, 5]. This is due to region-specific risk factors such as consumption of undercooked fish, liver fluke infestation (*Opisthorchis viverrini*), and subsequent chronic inflammation of the liver and the bile ducts [5]. In the Western World, the major risk factors for development of BTC are primary sclerosing cholangitis (PSC) and hepatitis B and hepatitis C viruses (HBV and HCV) as well as malformations of the biliary tract. In addition, lifestyle risk factors such as alcohol consumption, smoking, obesity, or exposure to certain chemicals and toxins contribute to the development of BTC and might at least in part explain the raising incidences in Europe and the US [2, 7]. The prognosis of BTC patients is very poor: the median survival after diagnosis is 24 months, and the 5-year survival rate is only about 10% [6]. The only potentially curative therapeutic option is complete resection of the tumor. However, due to the lack of efficient follow-up therapies and high therapeutic resistance, tumor recurrence is the norm [8]. Even more problematic in management of BTC is the long presymptomatic phase of the tumor progression which combined with the lack of potent biomarkers makes early diagnosis as the prerequisite for curative resection very difficult [9]. As a consequence, most patients are often diagnosed at already advanced stage of the disease, only allowing for palliative treatment with best supportive care. Currently, a combination of cisplatin and gemcitabine is the standard chemotherapeutic option for palliative treatment of BTC, leading to a median survival of only about one year [10, 11]. For nonresectable hilar BTC, photodynamic therapy is established as a palliative therapeutic option yielding 15-month median survival [12–14]. It is therefore evident that identification of new therapeutic options and, especially, biomarkers is of utmost importance to improve the prognosis of patients with BTC.

MicroRNAs (miRNAs) are short (20–22 nucleotides) noncoding RNAs that act as posttranscriptional regulators of gene expression via direct interaction with protein-coding mRNAs, thereby controlling several important physiological processes, such as cell proliferation, apoptosis, and cell differentiation. Biogenesis of miRNAs involves several steps. First, the miRNA gene is transcribed by RNA polymerase II, resulting in a long primary miRNA [15]. Noteworthy, miRNA genes can be transcribed as single transcriptional unit as well as polycistronically [16]. Then, the long primary miRNA transcripts are processed by the nuclear RNase Drosha resulting in shorter precursor miRNAs of a length of approximately 60 to 70 nucleotides [17]. Next, these precursors are exported from the nucleus to the cytoplasm, where they are cleaved by another RNase, called Dicer, to double-stranded miRNAs [18]. For regulation of mRNA transcripts, one strand of the mature miRNA is then incorporated in the RNA-induced silencing complex (RISC). In general, miRNAs exert their regulatory function by sequence-specific binding of their 5′ end, called the seed region, to the 3′ untranslated region (3′ UTR) of target mRNAs. Perfect match between the seed region and the 3′ UTR leads to degradation of the mRNA, whereas imperfect match results in inhibition of translation [19]. It is speculated that more than half of the protein-coding genes fall within the regulation of miRNAs. Furthermore, one miRNA species may have up to hundreds of protein-coding mRNAs as potential targets [20]. Today, over 28,000 miRNA species are known (based on miRBase, http://www.mirbase.org/).

These facts underline the central and essential role of miRNAs in regulating the realization of genetic information. More than a decade ago, first publications described miRNAs as a RNA species that is relevant in cancer [21, 22]. Today it is clear that deregulated miRNA expression plays a major role in the development and progression of various types of cancer (for a detailed review, see [23]). MicroRNAs can act as both oncogenes and tumor suppressor genes. As oncogenes, they are overexpressed in tumors, leading to excessive degradation of mRNAs that are coding for tumor suppressor proteins. As tumor suppressive factors, miRNAs are underexpressed in tumors, resulting in insufficient degradation of their target mRNAs, which often code for oncogenes. Fulfilling both of these roles, miRNAs influence and contribute to various aspects and hallmarks of cancer such as migration and invasion, proliferation, cancer stemness, metabolism, therapeutic resistance, and angiogenesis [23–25].

## 2. MicroRNA and BTC

At present, a reasonable number of published studies describe such a major role of miRNA deregulation for BTC tumorigenesis. Comprehensive microarray screens revealed numerous deregulated miRNA species in BTC samples and BTC model systems [26–37]. In this review, however, we will concentrate on miRNAs that were not only shown to be deregulated in BTC tissues but also for which direct target genes were functionally identified and/or validated. An overview of the miRNAs discussed in this chapter is given in Table 1.

To generally clarify potential involvement of miRNAs in BTC, Shu et al. measured the expression of Drosha and Dicer and found significant downregulation of both enzymes in GBC tissue [38]. Since both enzymes are absolutely essential in the production of mature miRNAs, this observation (i) strongly suggests a principal role of miRNA deregulation in BTC and (ii) also might lead to the conclusion that, in general, the type of miRNA deregulation in BTC might be underexpression more often than overexpression. In fact, as shown in Table 1 and considering the currently available data, most deregulated miRNAs in BTC tissues show decreased expression when compared to their healthy counterparts. Underexpression of these miRNAs uniformly correlates directly to various unfavorable clinicopathological characteristics such as advanced clinical stage, enhanced lymph node and distant metastasis, poor differentiation of tumor cells, and poor disease-free and overall survival. Hence, these miRNAs may function as tumor suppressors and their underexpression subsequently leads to diminished negative regulatory control of transcripts that encode for oncogenes. However, miRNAs can also act as oncogenes and overexpression of these miRNA species leads to increased degradation of mRNAs that otherwise would be translated into proteins with various tumor suppressor functions. Consequently, overexpression of oncogenic miRNAs is also correlated with disadvantageous clinicopathological features. To get more insight into the functional role and direct targets of tumor suppressor and oncogenic miRNAs, several* in vitro* and* in vivo* experiments were conducted. As described in detail below, these studies validated predicted direct target genes of miRNAs by luciferase reporter assays, downstream expression analysis, and various miRNA overexpression and knockdown experiments.

### 2.1. Tumor-Suppressive miRNAs in BTC and Their Direct Targets

MicroRNA 34a has been described to be downregulated in BTC tissue versus nontumor tissue by two independent studies [39, 40]. Functional* in vitro* and* in vivo* studies showed that PNUTS (see Abbreviations for full gene names) is a direct target of miRNA-34a. PNUTS is a protein that regulates telomere length and its overexpression reduces telomere shortening as well as apoptotic events connected with telomere shortening [41]. Telomere shortening is a mechanism that naturally limits the number of cell divisions of healthy cells and is known to be deregulated in cancer cells. Interestingly, in BTC tissues with decreased miRNA-34a expression, also increased telomere length was found [39]. The signal transduction molecule SMAD4 was identified as an additional target of miRNA-34a in BTC and was recently found to have a tumor-promoting role in hepatocellular carcinoma [40, 42]. In another study, the regulatory component of the 26S proteasome, PSMD10, was described as a target of deregulated miRNA-605 expression in BTC and, interestingly, an inverse expression pattern of miRNA-605 and PSMD10 was observed [43]. In an* in vitro* model, the authors demonstrated that overexpression of the tumor suppressor miRNA-605 resulted in suppression of BTC cell proliferation and this effect was rescued by ectopic expression of PSMD10, suggesting a direct mechanistic connection between miRNA-605 and PSMD10 as a driver of BTC cancer cell proliferation. In line with these observations, PSMD10 was shown to promote cell cycle progression and proliferation of pancreatic cancer cells [44]. Of note, enhanced PSMD10 expression was found to be directly involved in ubiquitylation and degradation of p53, a key tumor suppressor gene, which is also known to play a role in BTC [6, 45].

Detachment of cells from the primary tumor and subsequent carving through the extracellular matrix to invade the angiolymphatic system to eventually form metastasis is a hallmark of cancer in general and likewise in BTC [46–48]. The term “epithelial-mesenchymal transition” (EMT) describes a complex process that is essential for formation of secondary tumors and in which epithelial cells lose their polarity and gain mesenchymal traits including the ability to detach from the (primary) tumor [49, 50]. By controlling cell adhesion and cell-cell contact, E-Cadherin is an important epithelial and anti-EMT marker [51]. SLUG is a transcription factor that directly represses E-Cadherin, thereby activating EMT [52]. Qiu et al. demonstrated that the expression of miRNA-204 was lowered in IHC tissue and inversely correlated with the expression of SLUG. They also showed increased incidences of lymph node metastasis in patients with diminished miRNA-204 and enhanced SLUG expression. In addition, they identified SLUG as a direct target of miRNA-204 explaining the inverse expression patterns in the tissue samples [53]. Another miRNA, whose downregulation especially in metastatic BTC specimens was demonstrated, is miRNA-214 [54]. Using an* in vitro* model of BTC, the authors demonstrated a direct inhibitory effect of miRNA-214 on cell metastasis and EMT phenotype. They show that inhibition of miRNA-214 resulted in increased expression of EMT promoter TWIST, accompanied by decrease of epithelial marker, E-Cadherin. Furthermore, they identified TWIST as a direct functional target of miRNA-214. The miRNA-200 family was also shown to participate in EMT and metastasis regulation by directly targeting E-Cadherin repressors [55]. In a miRNA microarray study, Peng et al. found members of the miRNA-200 family to be underexpressed in CC samples [56]. Furthermore, in a BTC mouse model, they showed that upregulation of miRNA-200 family members resulted in inhibition of distant metastasis, underlining the role of this miRNA family in formation of secondary tumors. Searching for possible direct targets of miRNA family, they found ROCK2 to be a target of miRNA-200b/c. ROCK2 regulates cytoskeletal signaling events and cellular motility and was already shown to promote invasion of non-small cell lung cancer cells [57]. Of note, Peng et al. noticed overexpression of ROCK2 in CC samples, which, combined with the observed underexpression of miRNA-200 family members, strengthens the functional connection between these tumor suppressor miRNAs and the prometastatic factor ROCK2 [56]. In the same study, SUZ12 was also identified as a direct target of miRNA-200 family members. SUZ12 is a core component of the Polycomb Repressive Complex 2 (PRC2), a histone methyltransferase complex that performs a specific histone methylation, thereby influencing compaction of chromatin and ultimately access to the DNA and gene transcription. Recently, we described that PRC2 plays a role in development and progression of BTC [58, 59]. PRC2 is a master epigenetic regulator, and its general overactivation as well as overexpression of its core components influences several aspects of BTC carcinogenesis [58]. Peng et al. described that silencing of SUZ12 resulted in reduced anchorage-independent growth of CC cells and that regulation of SUZ12 by miRNA-200 family members therefore is important in BTC [56].

HMGA2 is a protein that also participates in chromatin-dependent regulation of gene activity by modifying the chromatin structure [60]. Overexpression of HMGA2 can serve as a predictor of poor prognosis in IHC [61]. In a study by Zhou and coworkers, HMGA2 was identified to be a direct target of tumor suppressor miRNA-26a, a miRNA species that was found to be downregulated in GBC tissue [62]. The same study has demonstrated that HMGA2 counteracts the antitumor effects of ectopic miRNA-26a expression, which characterizes HMGA2 as a miRNA-26a-regulated oncogene in BTC.

Besides histone modification, DNA methylation is another important mechanism of epigenetic gene regulation. In this context, Chen et al. described MBD2 as a direct target of miRNA-373, a tumor miRNA species downregulated in hilar CC, which was associated with advanced clinical stage [63]. MBD2 is involved in the DNA methylation-dependent repression of gene transcription as a reader of cytosine methylation and was suggested as a marker associated with poor prognosis for patients with hepatocellular carcinoma [64, 65]. MicroRNA-144 is another miRNA species found to be downregulated in CC tissues compared to nonmalignant tissues as well as in CC cell lines versus nonmalignant cells [66]. Although no clinicopathological features that are associated with deregulated miRNA-144 expression were presented in this study, the authors clearly demonstrate an inverse expression pattern of miRNA-144 (low expression) and LIS1 (high expression) in CC patient samples. Interestingly, another study described LIS1 as a driver of cell migration and invasive potential of lung cancer cells [67]. In line with this study, Yang et al. demonstrated that LIS1 is a direct target of miRNA-144 in BTC and, furthermore, they showed that ectopic expression of miRNA-144 diminished LIS1 expression [66]. Combined with their observation that knockdown of LIS1 reduced invasion of CC cells, miRNA-144 is yet another miRNA whose downregulation in CC can result in enhanced metastatic potential. Investigating the role of HCV in development of BTC, Zeng et al. observed diminished levels of miRNA-124 in IHC patient samples [68]. Ectopic expression of miRNA-124 resulted in reduced migration and invasiveness in CC cells as well as in reduced protein levels of SMYD3 which they identified as a direct target of miRNA-124. SMYD3 is a histone methyltransferase already shown to promote invasion of cancer cells [69]. It is worth mentioning that one of the downstream targets of SMYD3 is the metalloproteinase MMP-9, an enzyme that plays a pivotal role in the process of invasion by degrading the extracellular matrix [69]. By overexpressing miRNA-124, Zeng et al. observed downregulation not only of SMYD3 itself but also of its downstream target MMP-9 in CC cells [68].

Chemoresistance is a major problem in management of BTC [2]. In a recent study on GBC by Zhan et al., a correlation between miRNA-145 expression and sensitivity towards the standard chemotherapeutic cisplatin was shown [70]. Overexpression of miRNA-145 increased efficacy of cisplatin treatment, whereas lower levels of miRNA-145 decreased sensitivity towards cisplatin treatment in an* in vitro* model of BTC. Expression analysis of miRNA-145 in GBC tissue versus corresponding noncancerous gallbladder tissue showed downregulation of miRNA-145. As a direct target of miRNA-145, the authors identified MRP1, a family member of the “ATP Binding Cassette” drug efflux pumps [70]. These proteins are often upregulated in cancer cells and contribute to multidrug resistance in various types of cancer including BTC [59]. Zhan et al. recognized high sensitivity of miRNA-145 expressing tumors to cisplatin in a BTC mouse model, potentially caused by negative transcriptional control of MRP1 expression. By correlating expression data of miRNA-145 and MRP1 versus clinicopathological features of GBC patients that received chemotherapy, they noted that low expression of miRNA-145 is indeed linked to high expression of MRP1 and poor prognosis [70]. Of note, profiling of general miRNA expression might be a predictor of therapeutic efficiency/resistance as shown in our recent study on the mechanisms of photodynamic therapy in BTC cell lines [71]. In another study, Zhou and coworkers identified miRNA-135a-5p as an underexpressed miRNA species in GBC tissue and a negative correlation with VLDLR expression in the tested specimens [72]. VLDLR is a member of the low-density lipoprotein receptor superfamily which is involved in receptor-mediated endocytosis of specific ligands and has been reported to play a role in pathogenesis and tumor cell proliferation [73, 74]. Ectopic expression of miRNA-145 in GBC cells inhibited GBC cell growth* in vitro* and* in vivo* and this effect was VLDLR-dependent [72]. The metabolic profile of cancer cells differs significantly from their healthy counterparts. One well known phenomenon observed in the metabolism of cancer cells is that cancer cells favor aerobic glycolysis as primary ATP source instead of the far more efficient oxidative phosphorylation (“Warburg effect”) [75]. PKM2 is a rate-limiting enzyme that catalyzes the last step of glycolysis in a way that supports aerobic glycolysis. Unsurprisingly, PKM2 was found to be overly expressed in numerous cancer types [76]. Lu et al. identified PKM2 as a direct target of miRNA-122 in GBC cells. They showed further that, in GBC patient samples, miRNA-122 is underexpressed, whereas PKM2 expression is enhanced [77].

Another downregulated miRNA in BTC tissue is miRNA-138 as presented by Ma and coworkers [79]. Interestingly, they found a significant inverse correlation between miRNA-138 and BAG-1 expression in GBC tissue versus adjacent nonneoplastic tissue and furthermore identified BAG-1 as a direct target of miRNA-138. BAG-1 is a known antiapoptotic protein and silencing of BAG-1 in an* in vitro* model of BTC leads to apoptotic events [79, 99]. Of note, overexpression of miRNA-138 resulted in inhibition of GBC cell growth, whereas simultaneous overexpression of BAG-1 reversed this growth inhibitory effect, thus underlining the functional connection between miRNA-138 and its direct target BAG-1 [79].

### 2.2. Targets of Oncogenic MicroRNAs in BTC

MicroRNA-21 is a classic oncogenic miRNA species that contributes to carcinogenesis in various tumor types [23]. Regarding BTC, several studies found an overexpression of miRNA-21 in BTC tissue and a correlation with disadvantageous clinicopathological features such as poor disease-free and overall survival, advanced clinical stage, poor cell differentiation, and lymph node metastasis [82–85]. Due to its role as an overexpressed oncogenic miRNA, targets of miRNA-21 are often tumor suppressor genes. In BTC, several direct targets of miRNA-21 have been described. PTEN is a well-known tumor suppressor gene that is often mutated in cancer and was already functionally connected to development of GBC [58, 100, 101]. Wang et al. identified PTEN as a direct target of miRNA-21 in BTC [84]. To determine the clinical significance of this result, they performed Kaplan-Meier analyses and could demonstrate that high miRNA-21 correlated with poor disease-free and overall survival, whereas high PTEN expression correlated with enhanced disease-free and overall survival. Of note, PTEN was also identified as a direct target of two other oncogenic miRNAs in BTC, namely, miRNA-221 and miRNA-92a, which belong to the 17–92 cluster [87, 90]. Another direct target of miRNA-21 is 15-PGDH, an enzyme that converts prostaglandin E2 (PGE2) to its inactive form [82, 102]. BTC often develops under inflammatory conditions [6]. PGE2 is the primary prostaglandin that is involved in inflammation in various pathogenic processes and deregulated 15-PGDH activity results in diminished PGE2 conversion and subsequently in a more inflammatory environment that facilitates carcinogenesis [102, 103]. As a consequence, 15-PGDH acts as a tumor suppressor gene as already shown for breast cancer [104]. In the study by Lu et al., they showed not only that miRNA-21 is overexpressed in CC tissue, but also that 15-PGDH is a direct target of miRNA-21 and that PGE2 drives tumorigenesis in an BTC* in vitro* model [82]. As already mentioned, degradation of extracellular matrix by metalloproteinases is a key step in development of secondary tumors. TIMP3 is an inhibitor of MMP-9, a metalloproteinase that not only was identified in BTC as a downstream target of the potentially deregulated oncogene SMYD3 [68] but also whose enhanced expression was directly correlated to poor overall survival in patients with hilar CC [105]. Interestingly, TIMP3 is another direct target of oncogenic miRNA-21, and, in addition, overexpression of miRNA-21 in CC samples is correlated with diminished levels of TIMP3 in CC specimens [83]. Two studies described PDCD4 as an additional direct miRNA-21 target in BTC [83, 85]. PDCD4 is a tumor suppressor gene that regulates various aspects of carcinogenesis in different types of cancer and whose downregulation or loss is associated with poor prognosis [106–108]. Another direct target of miRNA-21 in BTC is PTPN14 [84], which was shown to be a negative regulator of metastasis as well as of the potentially cancer-driving Hippo/YAP pathway [109, 110].

Besides miRNA-21, other oncogenic miRNA species and their direct targets were also identified in BTC. Zhang et al. described miRNA-26a as a direct regulator of GSK-3*β*, which itself is a negative regulator of *β*-Catenin signaling [86]. Accumulation of the transcription factor *β*-Catenin in the nucleus is a consequence of an active WNT signaling pathway [111–113], leading to activation of diverse downstream genes. GSK-3*β* is part of the *β*-Catenin destruction complex, which, in the absence of active WNT pathway signaling, marks *β*-Catenin for proteasome-mediated destruction [114]. In their study, Zhang and coworkers observed increased miRNA-26a expression in CC tissues and cell lines [86]. Functional cell-based experiments demonstrated direct interaction between miRNA-26a and GSK-3*β*; in addition, miRNA-26a-mediated reduction of GSK-3*β* resulted in activation of *β*-Catenin and expression of cancer-driving genes such as cell cycle promoters. Another upregulated oncogenic miRNA in GBC was found by Chang and coworkers [81]. In this study, elevated miRNA-20a expression in GBC tissue was correlated with local invasion and distant metastasis. A functional explanation of this observation is given by the identification of SMAD7 as a direct target of miRNA-20a as well as the inverse expression pattern of miRNA-20a and SMAD7. High expression of miRNA-20a correlated with low expression of SMAD7 and this expression pattern resulted in very poor overall survival [81]. SMAD7 was first described as an inhibitor of TGF-*β* signaling but is now known as a versatile regulator of various signaling pathways. The role of SMAD7 in cancer progression is not uniform, as overexpression of SMAD7 can lead to both favorable and poor prognoses [115]. Interestingly, Huang et al. observed enhanced invasion and migration of GBC cells after SMAD7 inhibition, suggesting a more tumor suppressive role in this context, whereas, in another publication regarding BTC, SMAD7 was found to be overexpressed in CC samples and correlated with lower disease-free and overall survival [116].

Taken together, these studies show that deregulation of miRNAs plays a central role in development and progression of BTC and also translates into real clinical consequences. Overexpression or underexpression of certain miRNA species is associated with disadvantageous clinicopathological characteristics and poor disease-free and overall survival in BTC. The presented studies clearly demonstrate that individual miRNAs do act as tumor suppressors or oncogenes in BTC, depending on their actual regulatory targets. Of note, current evidence suggests that the same miRNA species can be both tumor-promoting and tumor-repressing within BTC. In the studies summarized in Table 1, miRNA-26a was found to be upregulated acting as an oncogenic miRNA, whereas it was found to be downregulated in another study, where it acted* de facto* as a tumor suppressor miRNA [62, 86]. Another example of this phenomenon is miRNA-200c, which was downregulated in CC samples in the study conducted by Peng et al. [56] but significantly upregulated in another study [117].

### 2.3. Regulation of miRNA Expression

Based on the presented studies, it is evident that deregulation of miRNAs influences various aspects of BTC carcinogenesis. Understanding the reasons of miRNA deregulation might therefore be of great scientific and clinical interest. As already described, miRNAs genes are transcribed by RNA polymerase II [15]. Like protein-coding genes, miRNA genes can be regulated by epigenetic events, altering the transcriptional accessibility of these genetic loci. DNA methylation at CpG islands in promoter regions is an epigenetic mechanism that leads to transcriptional repression of the respective gene. Chen et al. observed downregulation of miRNA-373 in patients with hilar cholangiocarcinoma [63]. In order to investigate the mechanism of the miRNA-373 deregulation, they analyzed the genomic surrounding of the miRNA-373 gene and identified a region at the 5′ flank that may serve as a promoter. Of note, this putative promoter harbors a potential CpG island, allowing for epigenetic regulation. Accordingly, analysis of the methylation status of this CpG island revealed high methylation rates, including homozygous methylation. Furthermore, high DNA methylation at this genomic region correlated with low miRNA-373 levels in BTC patient samples, suggesting that downregulation of miRNA-373 is a consequence of aberrant epigenetic regulation.

Another study that connects deregulated miRNA expression with DNA methylation was conducted by Zeng et al. [68]. Here, the authors investigated if the observed low expression of miRNA-124 in BTC samples is caused by enhanced DNA methylation. Inhibition of DNA methyltransferases significantly raised miRNA-124 expression. It is worth mentioning that this study was done using HCV-related IHC specimens and it is known that HCV infection is a major risk factor for development of BTC [2]. In this regard, Zeng et al. showed that HCV directly causes upregulation of DNA methyltransferases which in consequence leads to enhanced DNA methylation events and silencing of genes including miRNA-124 [68]. Further evidence of a central contribution of DNA methylation in regulation of miRNAs in BTC was presented by An et al. in a study investigating the underexpression of miRNA-370 in CC tissues [80]. Additionally, DNA demethylation in CC cells resulted in enhanced expression of miRNA-370. Looking at the imprinting status of miRNA-370, they found that the paternal allele of miRNA-370 was silenced via genomic imprinting, while the maternal allele was responsible for expression of this miRNA species. Therefore, reduction of miRNA-370, as observed in this study, has to be an implication of silencing of the maternal allele. An et al. also described overexpression of Interleukin-6 (IL-6) in their CC sample cohort, a cytokine that is well known to be a potent mitogen as well as a major proinflammatory factor in CC [118], and found that IL-6 induces DNA hypermethylation, thereby effectively suppressing the expression of miRNA-370 from the nonimprinted maternal allele [80]. This observation goes in line with another study that also described IL-6 as a promoter of DNA methylation, again leading to downregulation of the tumor suppressor miRNA-370 [119].

An interesting regulatory mechanism for miRNAs was described by Ma et al., which involves HOTAIR, a long-noncoding RNA which they found to be overly expressed in GBC patient material [120]. Silencing of HOTAIR led to an upregulation of miRNA-130a, suggesting a direct regulatory connection at the posttranscriptional level. Interestingly, this upregulation was only observed for mature miRNA-130a and not for its precursor forms. More evidence of direct regulation of miRNA-130a by HOTAIR relates to the fact that both of these RNA species were found to be present in the same RISC complex [120]. The potential oncogenic role of HOTAIR in BTC was already described for breast and colon cancer as well as for BTC [59, 121, 122].

## 3. MicroRNAs as Biomarkers

As mentioned in the introduction, one major problem in management of BTC is the advanced stage at time of diagnosis, excluding surgery as the only potentially curative treatment option [9]. Specific biomarkers in early stages of BTC would therefore allow rapid diagnosis and broaden the spectrum of therapeutic options available at time of diagnosis, which in turn should improve prognosis and outcome for patients with BTC. A suitable biomarker should be noninvasive, stable in fluids such as blood or bile, disease-specific, and easy to access and to measure and has to be sensitive in order to identify positive cases (high true positive rate) as well as being specific in order to distinguish positive from negative cases (low false positive rate) [123]. A common test for evaluation of a potential biomarker is the “Receiver Operating Characteristic” (ROC) analysis, in which the true positive rate and the false positive rate of the potential biomarker are plotted. The “area under the curve” (AUC) is then used for interpretation of the diagnostic power of the tested biomarker candidate. By definition, a perfect biomarker results in an AUC of 1.0, whereas a random chance results in an AUC of 0.5 [124, 125]. The currently employed biomarkers for BTC are carcinoembryonic antigen (CEA) and CA19-9, both showing rather inferior sensitivity and specificity [126, 127]. Since the discovery that miRNAs are stable in serum and plasma and that these circulating miRNAs can serve as specific biomarkers for various types of cancer, scientists worked on the identification of miRNA expression patterns that can be used as biomarkers for BTC [128]. For BTC, bile might also be an attractive body fluid for identification of diagnostic miRNAs, as potential biomarkers may be directly secreted by malignant cholangiocytes into bile and, therefore, samples may be more meaningful and specific due to the spatial proximity of the tumor cells and this matrix [127]. Accordingly, Li et al. proved the existence of diverse miRNA species in extracellular vesicles from human bile and demonstrated high stability of miRNAs expressed in biliary extracellular vesicles [129]. In the following paragraph, we will give an overview of studies that looked for specific miRNA expression patterns in order to potentially identify biomarkers regarding BTC (see Table 2).

Circulating miRNA-106a was downregulated in serum of CC patients compared to patients with benign biliary disease (BBD) and healthy individuals, correlating with poor prognosis and lymph node metastasis [91]. Of note, a gradual decline of miRNA-106a serum levels was observed, with highest expression in healthy individuals, medium expression in BBD, and lowest expression in CC patients. The authors then evaluated the diagnostic power of circulating miRNA-106a for discrimination of CC patients versus healthy individuals and CC patients against BBD. Serum miRNA-106a was very effective in distinguishing CC from nonmalignant cases, resulting in an AUC of 0.89, and moderate in distinguishing CC from BBD with an AUC of 0.79. Compared to the standard BTC biomarker, CA19-9, the authors concluded that serum miRNA-106a shows moderate-to-superior diagnostic value [91].

As discussed above, miRNA-21 is a proven and potent oncogene in BTC carcinogenesis (see Table 1). In addition, several studies investigated the diagnostic power of this miRNA species. In plasma samples, miRNA-21 was overexpressed in BTC compared to BBD and healthy controls and, interestingly, after surgery, plasma levels of miRNA-21 significantly declined [93]. Again, an expression gradient was observable: highest expression of plasma miRNA-21 in BTC patients, medium in BBD patients, and lowest in healthy individuals. MicroRNA-21 was superior over CA19-9 in differentiating BTC patients versus healthy individuals with sensitivity of 84% (CA19-9: 36.2%). However, combination of plasma miRNA-21 and CA19-9 levels grouped as a diagnostic panel resulted in even better sensitivity (90.4%). A similar result was seen for discrimination between patients with BTC and BBD, where miRNA-21 showed approximately twice the sensitivity of CA19-9 (71.2% versus 36.1%), but, again, combination of both factors resulted in overall better sensitivity (79.8%) [93]. Further evidence that qualifies secreted miRNA-21 as a potential biomarker comes from a study conducted by Wang et al., in which they measured serum levels of miRNA-21 in patients with IHC compared to healthy individuals [84]. Serum miRNA-21 was significantly enhanced in IHC samples and, in line with the study by Kishimoto and coworkers [93], miRNA-21 markedly declined after surgery. However, this effect was only observable for potentially curative surgery and not for palliative resection [84]. Again, miRNA-21 showed robust characteristics as a biomarker for BTC: serum miRNA-21 levels discriminated IHC patients from healthy individuals with high sensitivity (87.8%) and specificity (90.5%) and an AUC of 0.908. In urine, miRNA-21 levels were increased in periductal fibrosis and CC patients compared to healthy individuals. Micro-RNA-21 was able to distinguish healthy individuals from periductal fibrosis (AUC 0.735) and CC (0.820) patients [98]. Interestingly, the authors also observed enhanced levels of miRNA-192 in urine samples of* Opisthorchis viverrini*-infected patients as well as in periductal fibrosis and CC patients compared to healthy controls. The diagnostic power of miRNA-192 alone is moderate:* Opisthorchis viverrini*-infected (AUC 0.766); periductal fibrosis (0.781); CC (0.682) versus nonmalignant controls. However, combination of miRNA-21 and miRNA-192 in a biomarker panel enhanced their ability to discriminate healthy individuals from* Opisthorchis viverrini*-infected (AUC 0.812), periductal fibrosis (0.815), and CC (0.849) cases, making urine another possible noninvasive source of CC biomarkers [98]. Complementing these promising results in terms of miRNA-21 as a potential biomarker, Selaru et al. evaluated the diagnostic power of miRNA-21 expression in CC tissue and, although this study cannot contribute to the evaluation of miRNA-21 as a noninvasive diagnostic marker, the results are still valuable [83]. Selaru and coworkers saw not only significant overexpression of miRNA-21 in BTC tissue compared to nonmalignant control samples but also outstanding and nearly optimal sensitivity (95%), specificity (100%), and AUC (0.995) for miRNA-21 in distinguishing CC from normal bile duct [83].

Besides miRNA-21, serum miRNA-26a was also suggested to provide accuracy as a diagnostic tool for BTC [94]. Levels of miRNA-26a were increased in serum of CC patients compared to healthy control subjects and correlated with short progression-free and overall survival. Importantly, serum miRNA-26a was able to differentiate between patients with CC and healthy individuals with sensitivity and specificity values above 80% and an AUC of 0.899, thereby being a superior biomarker compared to CA19-9, which only displayed an AUC of 0.723. Of note, serum levels of miRNA-26a declined significantly in patients who underwent potential curative surgery [94]. In a large screen for differentially expressed miRNAs in serum of patients with BTC or PSC versus healthy individuals, Bernuzzi et al. identified miRNA-200c as being deregulated in PSC with an AUC value of 0.74 for distinguishing PSC from healthy control [95]. For BTC, they found serum levels of miR-483-5p and miR-194 to be enhanced compared to both PSC and controls (with control samples displaying the lowest expression levels). ROC curve analysis for both of these miRNA species resulted in AUC of 0.77 for miRNA-483-5p and AUC of 0.74 for miRNA-194. However, combination of these two miRNAs significantly increased the AUC value to 0.81 [95]. MicroRNA-192, which was already found to be upregulated in urine samples of CC patients as mentioned above [98], is another circulating miRNA of potential diagnostic value as published by Silakit and others [96]. This miRNA species was found to be upregulated in CC serum samples versus serum of healthy control subjects and associated with disadvantageous clinicopathological characteristics. In distinguishing CC patients from control individuals, miRNA-192 achieved sensitivity of 74%, specificity of 72%, and an AUC of 0.803 [96]. A similar AUC (0.791) was calculated for circulating miRNA-150 in plasma to differentiate between IHC patients and healthy controls and, compared to the calculated AUC of CA19-9 (0.747), the potential of plasma miRNA-150 was superior [26]. Again, a combination of plasma miRNA-150 and CA19-9 was significantly more powerful as a diagnostic tool for BTC than either factor alone, resulting in an AUC of 0.92 [26]. Concerning bile as a potential source of BTC biomarkers, Shigehara and coworkers confirmed presence and stability of endogenous miRNAs in bile, making bile a potentially attractive source of biomarkers for BTC, and therefore compared in a comprehensive microarray study the expression levels of miRNAs in bile of patients with BTC with those of patients suffering from BBD [97]. They found numerous deregulated miRNAs, including three that were markedly upregulated in BTC versus BBD: miRNA-9, miRNA-145^*∗*^, and miRNA-944. For miRNA-9 and miRNA-145^*∗*^, the outstanding AUC value of 0.975 suggests high potential as specific markers to discriminate BTC from BBD. The prognostic power of miRNA-944 was lower, albeit still resulting in an AUC value of 0.765 [97]. In another study using bile as source of putative diagnostic relevant miRNA species, Voigtländer et al. performed a large screen in bile and serum of patients with PSC and CC and observed different miRNA profiles between both diseases [92]. In serum, they found five upregulated miRNAs in PSC versus CC which showed good-to-moderate diagnostic power in distinguishing PSC from CC: miRNA-1281, miRNA-126, miRNA-26a, miRNA-30b, and miRNA-122. In bile, potential biomarker miRNAs that were able to distinguish the two diseases were downregulated in PSC versus CC: miRNA-1537, -412, -640, and -3189. Of note, AUC values of each of those four candidates were relatively equal, around 0.80 [92].

## 4. Discussion and Outlook

**Figure 1:**
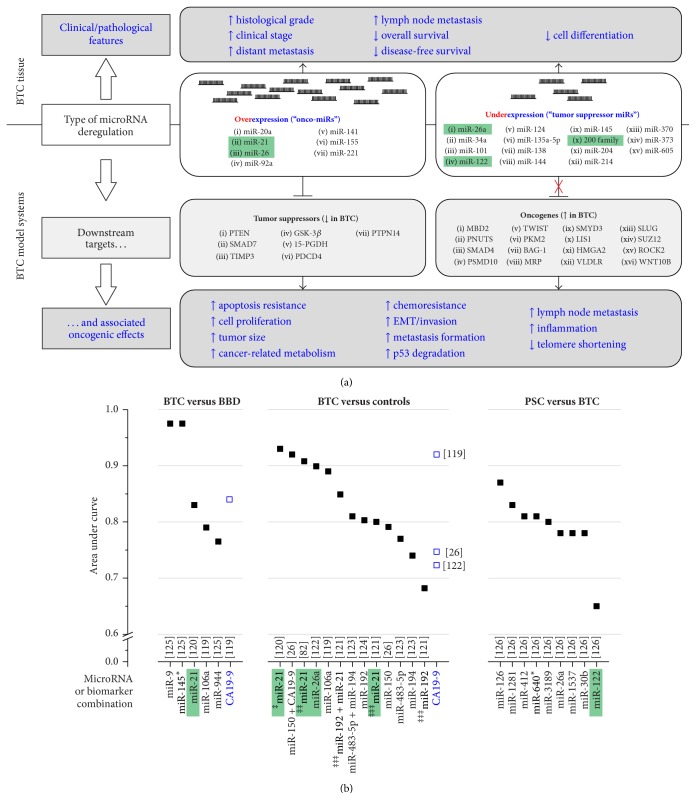
Role of deregulated miRs in BTC and their potential use as biomarkers. (a) Deregulation of miR expression in BTC tissue versus healthy controls results in unfavourable clinicopathological characteristics as well as poor outcome (upper part). Validated direct targets of deregulated miRs in BTC model systems include known tumor suppressors and oncogenes. Overexpression of oncogenic miRs results in aberrant downregulation of target tumor suppressors, whereas underexpression of tumor suppressor miRs results in insufficient negative transcriptional control of oncogenes (marked as red “X”), leading to their upregulation. Both of these events eventually cause diverse oncogenic effects (lower part). Figure based on Table 1. (b) Summary of distinguishing power of individual miRs regarding their use as potential biomarkers. The area under curve values from individual studies (reference numbers in square brackets; for details, see Table 2) indicate the quality of the respective miR as a biomarker (between 0.5 and max. 1.0), compared to CA19-9 as a conventional marker. Green boxes indicate miRs for which deregulation in BTC tissue as well as the use as a biomarker for BTC has been investigated. ‡: from plasma; ‡‡: from serum; ‡‡‡: from urine. For full gene names, see Abbreviations. BBD: benign biliary disease; BTC: biliary tract cancer; EMT: epithelial-mesenchymal transition; miR: microRNA; PSC: primary sclerosing cholangitis.

MicroRNAs are of great scientific and clinical interest, as it becomes more and more clear that this noncoding RNA species is a major factor in cancer diseases [23]. Their sheer number combined with their ability to potentially target multiple protein-coding transcripts gives an idea of the overall regulatory power of miRNAs [20]. For BTC, not only might miRNAs constitute new therapeutic targets themselves but also their function helps shed more light on the cellular and pathologic processes contributing to BTC carcinogenesis. As summarized in this review and in Figure 1(a), identification of direct targets of deregulated miRNAs in BTC can provide valuable knowledge about functional associations and new starting points for therapeutic strategies (Table 1). As one intensively investigated example, miRNA-21 was found to be frequently deregulated in BTC samples with poor clinicopathological features (Table 1). By directly promoting tumor growth and aggressiveness via direct transcriptional suppression of known tumor suppressor genes, miRNA-21 acts as a potent oncogenic miRNA.

Diagnosis of BTC at early stages is one important factor to improve prognosis. However, up to now, available biomarkers are not sensitive and specific enough [127]. MicroRNAs have been shown to circulate in a stable form in serum and plasma [128] and, in addition, are also detectable in bile fluid [129]. Therefore, several studies have already investigated expression patterns of miRNAs in plasma, serum, urine, and bile of BTC patients compared to healthy individuals. They found that circulating miRNAs obtained from body fluids have the potency to be sensitive and specific noninvasive biomarkers for BTC for diagnosing the tumor as well as for discriminating BTC from BBD (Table 2). Of note, the calculated diagnostic power of certain miRNAs exceeds the diagnostic power of the standard BTC marker, CA19-9, as illustrated in Figure 1(b). However, following the results of the presented studies, it may be useful to not just concentrate on one biomarker but rather combine a certain number of candidate biomarkers in a group to achieve maximum sensitivity, specificity, and diagnostic accuracy [26, 93, 95]. Again, for the mentioned miRNA-21, its aberrant expression in plasma, serum, urine, and tissue has superior diagnostic power in differentiating BTC from BBD and healthy controls, thus qualifying this miRNA as a potential biomarker (Table 2). In this regard, it will be interesting to determine the expression levels of miRNA-21 in bile of BTC patients and healthy patients to estimate a potential biomarker function also in this body fluid. Further, more detailed investigation of the role of miRNA-21 in early events of BTC development and carcinogenesis will be helpful to evaluate miRNA-21 as a biomarker for BTC.

## Figures and Tables

**Table 1:** MicroRNAs are deregulated in biliary tract cancer specimens.

	miRNA	Tissue	Clinicopathological characteristics associated with deregulated miRNA expression	Target	Ref.
Downregulated	26a	GBC	Advanced histologic grade	HMGA2	[62]
	34a	EHC, GBC	Poor disease-free and overall survival; increased telomere length; advanced clinical stage; lymph node metastasis	PNUTS, SMAD4	[39, 40]
	101	GBC	Enhanced tumor size; enhanced tumor invasion; higher TNM stage; poor survival		[78]
	122		Increased expression of PKM2	PKM2	[77]
	124	IHC		SMYD3	[68]
	135a-5p	GBC	Advanced histologic grade	VLDLR	[72]
	138		Increased expression of BAG-1	BAG-1	[79]
	144	CC	Increased expression of LIS1	LIS1	[66]
	145	GBC	Poor survival	MRP	[70]
	200 family	CC		SUZ12, ROCK2	[56]
	204	IHC	Lymph node metastasis	SLUG	[53]
	214		Enhanced metastatic potential	TWIST	[54]
	370	CC		WNT10B	[80]
	373	hCC	Poor cell differentiation; advanced clinical stage	MBD2	[63]
	605	IHC		PSMD10	[43]

Upregulated	20a	GBC	Local invasion; distant metastasis; poor prognosis and survival	SMAD7	[81]
	21	CC, IHC	Poor disease-free and overall survival; higher clinical stage at diagnosis; poor cell differentiation; lymph node metastasis	15-PGDH, PDCD4, TIMP3, PTPN14, PTEN	[82–85]
	26a	CC		GSK-3*β*	[86]
	92a			PTEN	[87]
	141	BTC	Shorter disease-free and overall survival; greater risk of angiolymphatic invasion		[88]
	155	GBC	Shorter disease-free survival; lymph node metastasis; vessel invasion		[89]
	221	EHC	Shorter disease-free survival; advanced clinical stage	PTEN	[90]

CC: cholangiocarcinoma; EHC: extrahepatic cholangiocarcinoma; GBC: gallbladder carcinoma; hCC: hilar cholangiocarcinoma; IHC: intrahepatic cholangiocarcinoma; miRNA: microRNA.

**Table 2:** MicroRNAs as potential biomarkers for BTC.

	miRNA (source)	Groups	Clinicopathological characteristics associated with deregulated miRNA expression	AUC	Ref.
Downregulated	106a (serum)	CC versus control	Lymph node metastasis; poor prognosis	0.89	[91]
	106a (serum)	CC versus BBD		0.79	[91]
	1537 (bile)	PSC versus PSC/CC		0.78	[92]
	412 (bile)			0.81	[92]
	640^*∗*^ (bile)			0.81	[92]
	3189 (bile)			0.80	[92]

Upregulated	21 (plasma)	BTC versus control	Decline of miRNA-21 plasma levels after surgery	0.93	[93]
	21 (plasma)	BTC versus BBD		0.83	[93]
	150 (plasma)	IHC versus control		0.791; 0.920 (+CA19-9)	[26]
	21 (tissue)	CC versus control		0.995	[83]
	21 (tissue)	IHC versus control	Decline of miRNA-21 serum levels after potentially curative surgery	0.908	[84]
	26a (serum)	CC versus control	Decline of miRNA-26a serum levels after potentially curative surgery; shorter progression-free and overall survival	0.899	[94]
	483-5p (serum)			0.77; 0.81 (+miRNA 194)	[95]
	194 (serum)			0.74; 0.81 (+miRNA 483-5p)	[95]
	192 (serum)		Lymph node metastasis; shorter survival	0.803	[96]
	200c (serum)	PSC versus control		0.74	[95]
	1281 (serum)	PSC versus CC		0.83	[92]
	126 (serum)			0.87	[92]
	26a (serum)			0.78	[92]
	30b (serum)			0.78	[92]
	122 (serum)			0.65	[92]
	9 (bile)	BTC versus BBD		0.975	[97]
	145^*∗*^ (bile)			0.975	[97]
	944 (bile)			0.765	[97]
	21 (urine)	CC versus control		0.820; 0.849 (+miRNA 192)	[98]
	192 (urine)			0.682; 0.849 (+miRNA 192)	[98]

AUC: area under curve; BBD: benign biliary diseases; BTC: biliary tract cancer; CC: cholangiocarcinoma; IHC: intrahepatic cholangiocarcinoma; miRNA: microRNA; PSC: primary sclerosing cholangitis; PSC/CC: cholangiocarcinoma complicating primary sclerosing cholangitis.

## Abbreviations

15-PGDH: Hydroxyprostaglandin Dehydrogenase 15-(NAD)
3′ UTR: 3′ untranslated region
AUC: Area under the curve
BAG-1: BCL2-associated athanogene 1
BBD: Benign biliary disease
BTC: Biliary tract cancer
CC: Cholangiocarcinoma
EHC: Extrahepatic cholangiocarcinoma
EMT: Epithelial-mesenchymal transition
GBC: Gallbladder cancer
GSK-3*β*: Glycogen synthase kinase 3 beta
HBV: Hepatitis B virus
hCC: hilar cholangiocarcinoma
HCV: Hepatitis C virus
HMGA2: High-mobility group AT-hook 2
IHC: Intrahepatic cholangiocarcinoma
LIS1: Platelet activating factor acetylhydrolase 1b regulatory subunit 1
MBD2: Methyl-CpG binding domain protein 2
miRNA: MicroRNA
MMP-9: Matrix metallopeptidase 9
MRP1: ATP Binding Cassette subfamily C member 1
PDCD4: Programmed cell death 4
PGE2: Prostaglandin E2
PKM2: Pyruvate kinase, muscle
PNUTS: Protein phosphatase 1 regulatory subunit 10
PRC: Polycomb Repressive Complex
PSC: Primary sclerosing cholangitis
PSMD10: Proteasome 26S subunit, non-ATPase 10
PTEN: Phosphatase and tensin homolog
PTPN14: Protein tyrosine phosphatase, nonreceptor type 14
RISC: RNA-induced silencing complex
ROC: Receiver Operating Characteristic
ROCK2: Rho associated coiled-coil containing protein kinase 2
SLUG: Snail family zinc finger 2
SMAD4: SMAD family member 4
SMAD7: SMAD family member 7
SMYD3: SET and MYND domain containing 3
SUZ12: SUZ12 polycomb Repressive Complex 2 subunit
TGF-*β*: Transforming growth factor-beta
TIMP3: TIMP metallopeptidase inhibitor 3
TWIST: Twist family BHLH transcription factor 1
VLDLR: Very low-density lipoprotein receptor
WNT10B: Wnt family member 10B
YAP: Yes-associated protein 1.
